# Congratulations to *Physiological Reports*

**DOI:** 10.14814/phy2.15875

**Published:** 2023-11-28

**Authors:** Susan Wray

**Affiliations:** ^1^ Department of Women's and Children's Health University of Liverpool Liverpool UK

When I was appointed as the first Editor‐in‐Chief of *Physiological Reports* (PR), the world of publishing was already very different from what I had grown used to during most of my research career. There was growing opposition to the model in which publication was funded by institutions subscribing to journals. This meant that only those with the funds required to subscribe could read the science, at least without waiting for an embargo period of 6 months or more. The Open Access movement argued that since research was generally funded by the public, it should be available for all to read.

*Physiological Reports* was established as a cooperative venture between the American Physiological Society (APS) and The Physiological Society (TPS). So, the Editor‐in‐Chief had two mistresses to serve! The societies had many years' experience of publishing, with *The Journal of Physiology* (JP) founded in 1878 and *The American Journal of Physiology* (AJP) in 1898. Neither Society, however, had published an Open Access journal. *Terra Nova*—exciting, challenging and at times, daunting.

*Physiological Reports* was also different from its better‐established sister journals in the way in which it received manuscripts. While some were submitted by the conventional route, a large fraction were “cascaded” from JP and AJP. These were scientifically sound manuscripts, but which had not met the criterion of impact demanded by those journals. This process demanded good links between PR and the other journals. I remember going to many APS Editorial Board meetings, trying to persuade the longer established journals that PR was indeed respectable, and being rewarded with doughnuts, submissions, and enduring friendships. *Acta Physiologica* became another supporting journal for cascading to PR and brought in good relations with the Scandinavian Physiological Society.

One frustration was the time it took for PR to be indexed on PubMed and other platforms. Indeed, it took three Editors‐in‐Chief and 10 years for it to be awarded the cherished Impact Factor. This does make me reflect on the power of the Impact Factor, an arbitrary metric relating to the number of citations garnered by the average paper in a 2‐year period after publication.

Another issue that is still evolving is the way in which academic journal publishing is funded. The traditional, subscription‐based model has been criticized, not only because of the requirement to pay to read, but also because the profits accrue to the publishers, and scientists give their time freely, as authors and reviewers. Of course, in the case of JP and APS, the publishers are scientific societies and the income supports their charitable activities. The move to Open Access poses a threat to such organizations, as subscription income falls are not matched by article processing charges. Another problem with Open Access is that it has replaced a system where one needs money to read, with one where money is required to publish, perhaps not as big an improvement as the founders of Open Access envisaged.

*Physiological Reports* and many other Open Access journals make a major positive contribution to the scientific landscape. The same cannot, however, be said for the outputs of several “predatory” publishers. Since Open Access is funded by authors, the more articles that are published, the more profit is made. In order to publish as many papers as possible, there may be little or no reviewing of the content. Thus, PR sometimes felt on the back foot, as some physiologists considered all open access journals tarnished.

The real success and achievements of PR, and its establishment in the ranks of good physiological journals, is down to the past and present dedicated editorial teams, the belief and support of APS and TPS, and the publisher Wiley, who all tirelessly promoted the journal and delivered on editorial promises of efficiency and transparency. In public and private conversations, I referred to PR as the “author's friend,” as we were not looking for reasons to reject their submissions. This has become an enduring characteristic of PR, and one that will contribute to its success in the next 10 years and beyond.

Happy birthday PR and congratulations to Jo and her team.

